# Abdominal aorta diameter as a novel marker of diabetes incidence risk in elderly women

**DOI:** 10.1038/s41598-020-70736-1

**Published:** 2020-08-13

**Authors:** Tadeusz Dereziński, Dorota Zozulińska-Ziółkiewicz, Aleksandra Uruska, Mariusz Dąbrowski

**Affiliations:** 1 Primary Care Clinic “Esculap”, Gniewkowo, Poland; 2 Department of Internal Medicine and Diabetology, Poznań University of Medical Sciences, Raszeja Hospital, Mickiewicza 2, 60-834 Poznan, Poland; 3 College of Medical Sciences, University of Rzeszów, Rzeszow, Poland

**Keywords:** Risk factors, Type 2 diabetes

## Abstract

The prevalence of diabetes mellitus is increasing worldwide, including the nation of Poland. The aim of this prospective and observational study was to determine risk factors and the predictors of diabetes incidence in elderly women, and to calculate the diabetes incidence ratio in this population. Two-hundred women, aged 65–74, who were non-diabetic at baseline in 2012 were followed for 6.5 years. All women were checked for incident diabetes. In non-diabetic subjects, diagnostic procedures for diabetes were performed according to Poland’s Diabetes recommendations. Between April 2012 and September 2018, 25 women developed diabetes and the next 11 cases were diagnosed based on FPG or oral glucose tolerance test. Women with incident diabetes had significantly higher baseline FPG, triglycerides (TG), TG/HDL cholesterol ratio and visceral adiposity index (VAI) score, and lower abdominal aorta diameter (AAD), HDL cholesterol and eGFR. In the Cox proportional hazard regression analysis, only AAD < 18 mm and VAI score ≥ 3.8 were independently associated with diabetes risk, hazard ratio (HR) 2.47 (95% confidence interval 1.21–5.02), *P* = 0.013 and HR 2.83 (1.35–5.94), *P* = 0.006 respectively. In the backward stepwise regression analysis including all variables, diabetes incidence could be predicted from a linear combination of the independent variables: AAD < 18 mm (*P* = 0.002), VAI score ≥ 3.8 (*P* < 0.001) and FPG ≥ 5.6 mmol/L (*P* = 0.011). The calculated incidence of diabetes was 2769.2 new cases/100,000 persons per year. AAD below 18 mm seem to be a novel, independent marker of diabetes risk in elderly women, and AAD assessment during routine abdomen ultrasound may be helpful in identifying females at early elderliness with high risk of diabetes incidence.

## Introduction

The rising tide of diabetes mellitus prevalence and incidence is being observed worldwide with 463 million people suffering from diabetes in the year 2019^1^. The same trend has also been observed in Poland. In the year 2014, 2.34 million people (6.08% of the total population) were treated with antidiabetic medications^2^. Type 2 diabetes is especially highly prevalent among people aged over 65 and roughly one third of all cases are diagnosed in this age range^1,3^. In South-Eastern Poland, its prevalence in 2018 among people aged over 65 reached 26% of population in this age range (data on request from National Health Fund, unpublished). According to data published by the National Health Fund and Central Statistical Office, diabetes incidence in the Kuyavian-Pomeranian Voivodeship (central Poland) in women aged > 65 was 1860.7 new cases per 100,000 persons per year, consisting 45.6% of all new cases of diabetes in 2016^4^.

Established risk factors of diabetes incidence include: family history of diabetes, increased BMI, abdominal obesity, low physical activity, hypertension, impaired glucose metabolism in the past, high-risk race/ethnicity, elevated triglycerides (TG) and/or low HDL cholesterol level, and, among women, delivering children with birth weight over 4000 g, a history of gestational diabetes mellitus (GDM), and having polycystic ovary syndrome (PCOS)^1,5,6^. In our study conducted in 2012 other factors significantly associated with diabetes prevalence were also found: anthropometric and metabolic indices, such as waist-to-hip ratio (WHR), weight-to-height ratio (WHtR), triglycerides to HDL cholesterol (TG/HDL) ratio and Visceral Adiposity Index (VAI), as well as impaired kidney function, and lower abdominal aorta diameter (AAD) (in newly diagnosed patients only)^7^.

The main objective of this prospective and observational study was to determine the risk factors and the predictors of diabetes incidence in women reaching early elderliness, and to verify our previous finding on whether lower AAD is associated with an increased risk of developing diabetes^7^. The secondary aim was to calculate the actual diabetes incidence in this population.

## Methods

### Study participants

**Figure 1:**
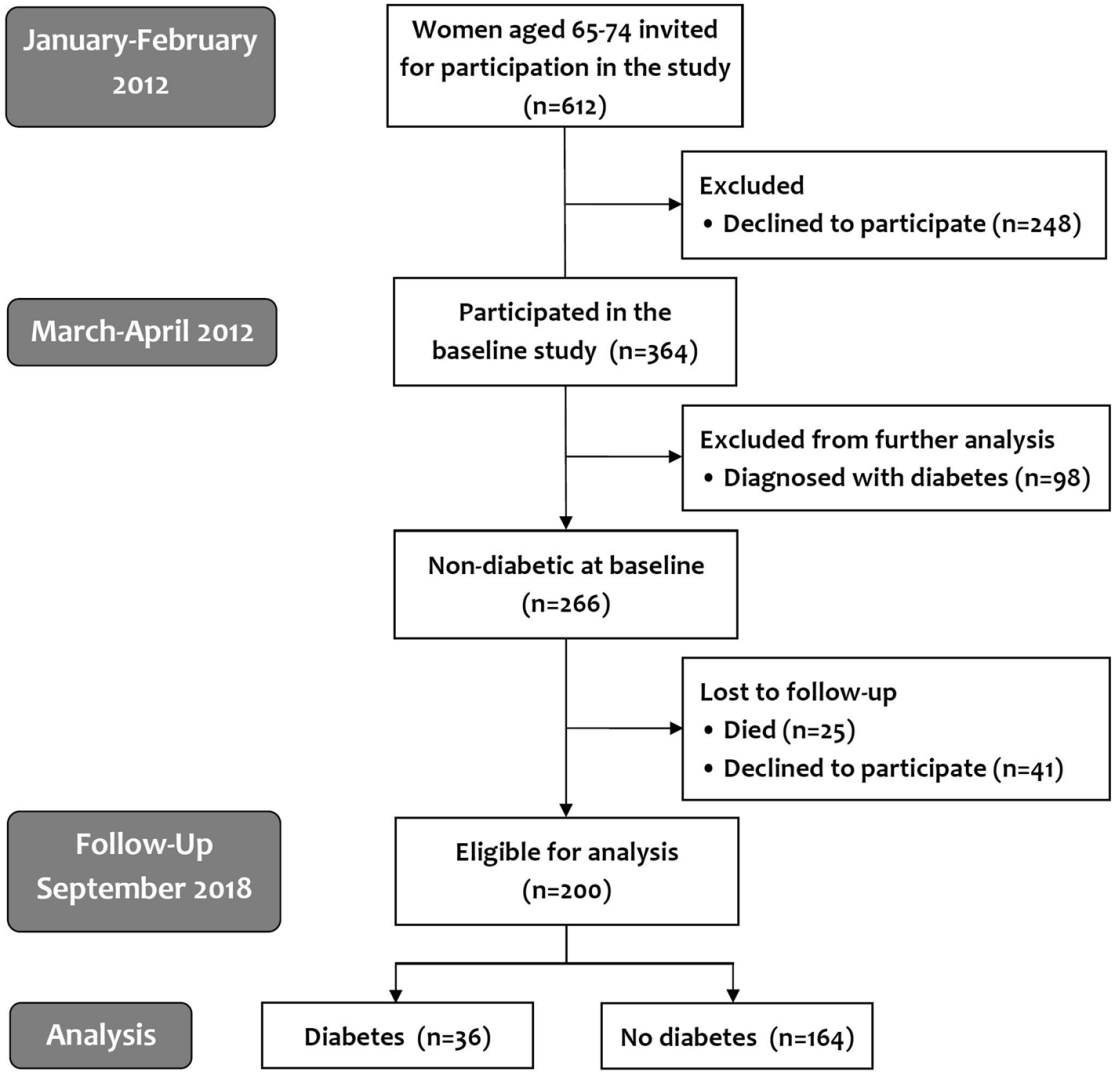
The study design and CONSORT flow diagram of study participants.

In March and April 2012, we conducted a study analyzing prevalence of diabetes and prediabetes in 364 women of Caucasian ethnicity, aged 65–74. At that time, all 612 women in that age range living in Gniewkowo, a community in central Poland with14,849 inhabitants in 2012 (7466 women)^8^, were invited to participate in the study. Of them, 364 (59.5%) responded to the invitation. The recruitment process is presented in Fig. 1. More details can be found elsewhere^7,9^. In that study, an analysis of associations between abnormal glucose metabolism and numerous anthropometric, metabolic and clinical factors was performed. In addition, abdominal aorta diameter and carotid intima/media thickness (CIMT) were measured. Abdomen ultrasound examination was performed by the same trained radiologist using an Aloka ProSound Alpha 6 device (Hitachi Aloka Medial Ltd., Tokyo, Japan) with a convex 3.5 MHz transducer. The aorta diameter was measured from the outer edge to the outer edge in the section between renal arteries to the bifurcation, and the highest value was taken for analysis. CIMT measurement was performed by another trained radiologist using a linear 5–13 MHz transducer. The evaluation was performed according to the Mannheim Carotid Intima-Media Thickness and Plaque Consensus, and the highest CIMT was used in the analysis^10^. Both radiologists had required certification.

Among screened females, 98 cases of diabetes (25 newly diagnosed) were found^7^. These females were excluded from the current analysis. After 6.5 years, a follow-up assessment of the remaining 266 women who were non-diabetic at baseline was performed.

Out of these 266 women, 25 died between April 2012 and September 2018. The remaining 241 women were invited for the follow-up visit. However, 41 of them declined to participate (Fig. 1). Among 200 females available for analysis (75.2% of all invited), 79 had impaired fasting glucose (IFG) and/or impaired glucose tolerance (IGT) at baseline, while 121 had normal glucose tolerance (NGT). Between April 2012 and September 2018, 25 women were determined to have diabetes and received treatment. In the remaining 175 women, blood samples for fasting plasma glucose (FPG) measurements were obtained. In females with FPG ≥ 7.0 mmol/L, the measurement was repeated, and in women with FPG within IFG range (5.6–6.9 mmol/L), an oral glucose tolerance test (OGTT) was performed. Diabetes, IFG and/or IGT were diagnosed according to Diabetes Poland criteria^6^. Apart from established risk factors of diabetes, we included into our analysis other factors associated with prevalent diabetes in 2012: AAD, pulse pressure, eGFR using CKD-EPI equation^11^, WHR, WHtR, TG/HDL ratio (calculated in mg/dL) and VAI^7^. VAI was calculated according to the formula developed by Amato et al. for women^12^:

$$ \begin{aligned} {\text{VAI}} & = \left[ {\frac{{{\text{WC}}}}{{36.58 + \left( {1.89{ } \times {\text{ BMI}}} \right)}}} \right] \times \left[ {\frac{{{\text{TG}}}}{0.81}} \right] \times \left[ {\frac{1.52}{{{\text{HDL}}}}} \right] \\ & {\text{TG}}\;{\text{and}}\;{\text{HDL}}\;{\text{cholesterol}}\;{\text{expressed}}\;{\text{in}}\;{\text{mg/dL}} \\ \end{aligned} $$

Moreover, the relationship between incident diabetes and tobacco smoking, statin and antihypertensive medications use were also analyzed.

### Statistical analysis

Statistical analysis of the data was performed using SigmaPlot for Windows, version 12.5 (Systat Software Inc., San Jose, CA, USA) and PQStat v.1.8.0 (PQStat Software, Poznań, Poland). All baseline continuous variables were summarized using descriptive statistics and they are presented as mean and standard deviation (SD). The normality of data distribution was checked using the Shapiro–Wilk test. Differences between the groups were analyzed using an unpaired two-tailed Student’s *t*-test or by a Mann–Whitney rank sum test where appropriate. Categorical variables were analyzed using χ^2^ test with the Yates continuity correction applied. Relationship between analyzed variables and incident diabetes was evaluated in the univariate and in the multivariate (Cox Proportional Hazard regression) models. Cut-off points for age, WHtR, AAD, TG/HDL ratio and VAI score were determined using the maximized sum of their sensitivity and specificity. Cut-off values for FPG (5.6 mmol/L), TG (1.7 mmol/L) and HDL cholesterol (1.3 mmol/L) were taken from the Diabetes Poland clinical practice recommendations^6^. A WC value ≥ 88 cm and WHR > 0.85, considered by World Health Organization (WHO) experts as cut-off points for substantially increased risk of metabolic complications in persons of European ethnicity, were taken^13^. To determine independent predictors of diabetes incidence, backward stepwise regression analysis was used. A *P* value of < 0.05 was assumed as statistically significant.

### Prior presentation

Parts of this study were presented as a poster at the 79th American Diabetes Association Scientific Sessions, San Francisco, CA, 7–11 June 2019, and they were published in form of abstract: *Diabetes.***68** (Supplement 1), 209-LB. (2019). https://doi.org/10.2337/Db19-209-Lb.

### Ethics approval and consent to participate

The study was approved by the Bioethics Committee at the Collegium Medicum in Bydgoszcz of the Nicolaus Copernicus University in Toruń, and it was conducted in accordance with ethical standards laid down in an appropriate version of the Declaration of Helsinki and in Polish national regulations. All study participants before beginning of the study procedures signed informed consent form.

## Results

Between April 2012 and September 2018, out of the 200 women who were non-diabetic at baseline, 25 females were diagnosed with diabetes. Another 11 cases were diagnosed upon FPG or oral glucose tolerance test in September 2018. Overall, at follow-up, 36 new cases of diabetes were identified. Among them, 25 had IFG and/or IGT in 2012. Calculated diabetes incidence rate was 2769.2 new cases per 100.000 persons a year, 95% confidence interval (CI) 2006.6–3821.6.

Women with incident diabetes had significantly higher baseline FPG, TG, TG/HDL ratio and VAI, and lower AAD, HDL cholesterol and eGFR. Other analyzed variables were not significantly different between the groups (Table 1).

**Table 1:** Differences between baseline values of analyzed variables among women with and without incident diabetes.

Parameter	Diabetes n = 36		No diabetes n = 164		*P* value
	Mean	SD	Mean	SD	
Age (years)	69.6	3.6	68.9	3.3	N.S
Height (cm)	158.2	6.7	157.1	6.3	N.S
BMI (kg/m^2^)	30.5	5.0	29.8	5.1	N.S
Waist circumference (cm)	99.2	11.1	95.3	12.4	N.S
Hip circumference (cm)	113.4	9.4	110.4	11.2	N.S
Waist/Hip Ratio (WHR)	0.87	0.06	0.86	0.07	N.S
Waist-to-Height Ratio (WHtR)	0.63	0.08	0.61	0.08	N.S
*Abdominal aorta diameter (AAD)* (mm)	*18.1*	*4.7*	*18.4*	*2.5*	*0.019*
*AAD/body surface area (BSA) ratio *(mm/m^2^)	*10.2*	*2.8*	*10.7*	*1.6*	*0.003*
Systolic blood pressure (mm Hg)	146.4	20.2	148.5	21.9	N.S
Diastolic blood pressure (mm Hg)	83.5	10.4	82.9	10.6	N.S
Pulse pressure (mm Hg)	62.9	15.0	65.6	16.6	N.S
Total cholesterol (mmol/L)	5.62	1.12	5.80	1.29	N.S
*HDL cholesterol* (mmol/L)	*1.52*	*0.33*	*1.78*	*0.45*	< *0.001*
Non-HDL cholesterol (mmol/L)	4.10	1.24	4.02	1.27	N.S
LDL cholesterol (mmol/L)	3.39	1.09	3.46	1.00	N.S
*Triglycerides* (mmol/L)	*1.61*	*0.62*	*1.31*	*0.56*	*0.002*
*Fasting plasma glucose* (mmol/L)	*6.03*	*1.00*	*5.40*	*0.61*	< *0.001*
*Triglycerides/HDL cholesterol ratio*^a^	*2.58*	*1.22*	*1.89*	*1.22*	< *0.001*
*Visceral adiposity index (VAI)*	*5.11*	*2.52*	*3.68*	*2.59*	< *0.001*
Creatinine (μmol/L)	69.0	14.1	63.6	14.1	N.S
*eGFR CKD-EPI* (mL/min/*1.73* m^2^)	*77.42*	*14.54*	*83.05*	*17.23*	*0.045*

Significant differences are in italics.

*SD* standard deviation, *N.S.* non-significant.

^a^Measured in mg/dL.

In the Framingham Heart Study, mean AAD in women aged ≥ 65 was related to body surface area (BSA). Females with BSA < 1.9 m^2^ had mean AAD 17.8 mm, while those with BSA ≥ 1.9 m^2^ had mean AAD 18.7 mm^14^. Our study participants with BSA < 1.9 m^2^ had a mean AAD of 18.3 ± 3.1 mm, while those with BSA ≥ 1.9 m^2^ had a mean of 18.6 ± 2.7 mm. After adjustment of AAD to BSA, the AAD/BSA ratio remained significantly lower in women with incident diabetes (Table 1). If women with abdominal aorta aneurysm were excluded, this difference became more pronounced, 9.7 ± 1.6 versus 10.7 ± 1.6, *P* < 0.001.

In univariate analysis WHtR > 0.65, AAD < 18 mm, TG ≥ 1.7 mmol/L, TG/HDL ratio ≥ 2.08, VAI ≥ 3.80, FPG ≥ 5.6 mmol/L and eGFR < 75 ml/min/1.73 m^2^ were significantly associated with diabetes incidence (Table 2).

**Table 2:** Hazard ratios (HR) of diabetes incidence across analyzed variables in univariate analysis.

Parameter	HR (95% CI)	*P* value
Age ≥ 69 years	1.38 (0.72–2.67)	N.S
BMI ≥ 25 kg/m^2^	1.23 (0.48–3.15)	N.S
Waist ≥ 88 cm	2.26 (0.80–6.38)	N.S
Waist/Hip Ratio (WHR) > 0.85	1.48 (0.74–2.96)	N.S
*Waist-to-Height Ratio (WHtR)* > *0.65*	*1.96 (1.02–3.78)*	*0.045*
Hypertension	2.00 (0.71–5.66)	N.S
*Abdominal Aorta Diameter (AAD)* < *18 *mm	*2.66 (1.33–5.32)*	*0.006*
HDL cholesterol ≤ 1.3 mmol/L	1.88 (0.91–3.89)	N.S. (0.090)
*Triglycerides ≥ 1.7* mmol/L	*2.26 (1.15–4.41)*	*0.017*
*Triglycerides/HDL cholesterol (TG/HDL) ratio*^a^	*3.29 (1.66–6.49)*	< *0.001*
*Visceral adiposity index (VAI)*	*3.56 (1.75–7.22)*	< *0.001*
*Fasting plasma glucose (FPG) ≥ 5.6* mmol/L	*2.89 (1.42–5.88)*	*0.003*
*eGFR CKD-EPI* < *75* mL/min*/1.73* m^2^	*2.11 (1.10–4.05)*	*0.025*

Significant associations in italics.

*eGFR* estimated Glomerular Filtration Rate.

^a^Measured in mg/dL.

When women with abdominal aorta aneurysm (n = 2) were excluded from the analysis, the risk of diabetes incidence for women with AAD < 18 mm became even higher, Hazard ratio (HR) 3.13 (95% CI 1.59–6.20, *P* < 0.001).

Women with incident diabetes had lower AAD irrespective of glucose metabolism status at baseline. Among 79 women with IFG/IGT at baseline, 25 developed diabetes, while 54 did not. Mean AAD in these groups was respectively 17.4 ± 2.4 mm versus 18.4 ± 2.1 mm, *P* = 0.034. Women with IFG/IGT and AAD < 18.0 mm had significantly elevated risk of incident diabetes, HR 2.29 (1.12–4.69), *P* = 0.030. Among women with NGT at baseline, when 2 females with abdominal aorta aneurysm were excluded, those who developed diabetes had a significantly lower AAD of 16.3 ± 2.3 mm compared to women who remained normoglycemic, 18.4 ± 2.7 mm, *P* = 0.028. HR was not calculated due to the small number of incident cases.

Tobacco smoking, use of statin and main classes of anti-hypertensive medications were not significantly different between the groups, and only thiazide diuretics were associated with borderline insignificantly elevated risk of diabetes incidence, HR 2.10 (0.96–4.60), *P* = 0.065 (Table 3).

**Table 3:** Tobacco smoking, statin and anti-hypertensive medications use among women with and without incident diabetes (all differences between groups are insignificant).

Parameter	Diabetes n = 36		No diabetes n = 164	
	n	%	n	%
Tobacco smoking	14	38.9	49	29.9
Statins	11	30.6	70	42.7
ACE inhibitors/sartans	21	58.3	89	54.3
Calcium channel blockers	11	30.6	33	20.1
Beta blockers	15	41.7	70	42.7
Thiazide diuretics	8	22.2	16	9.8

**Figure 2:**
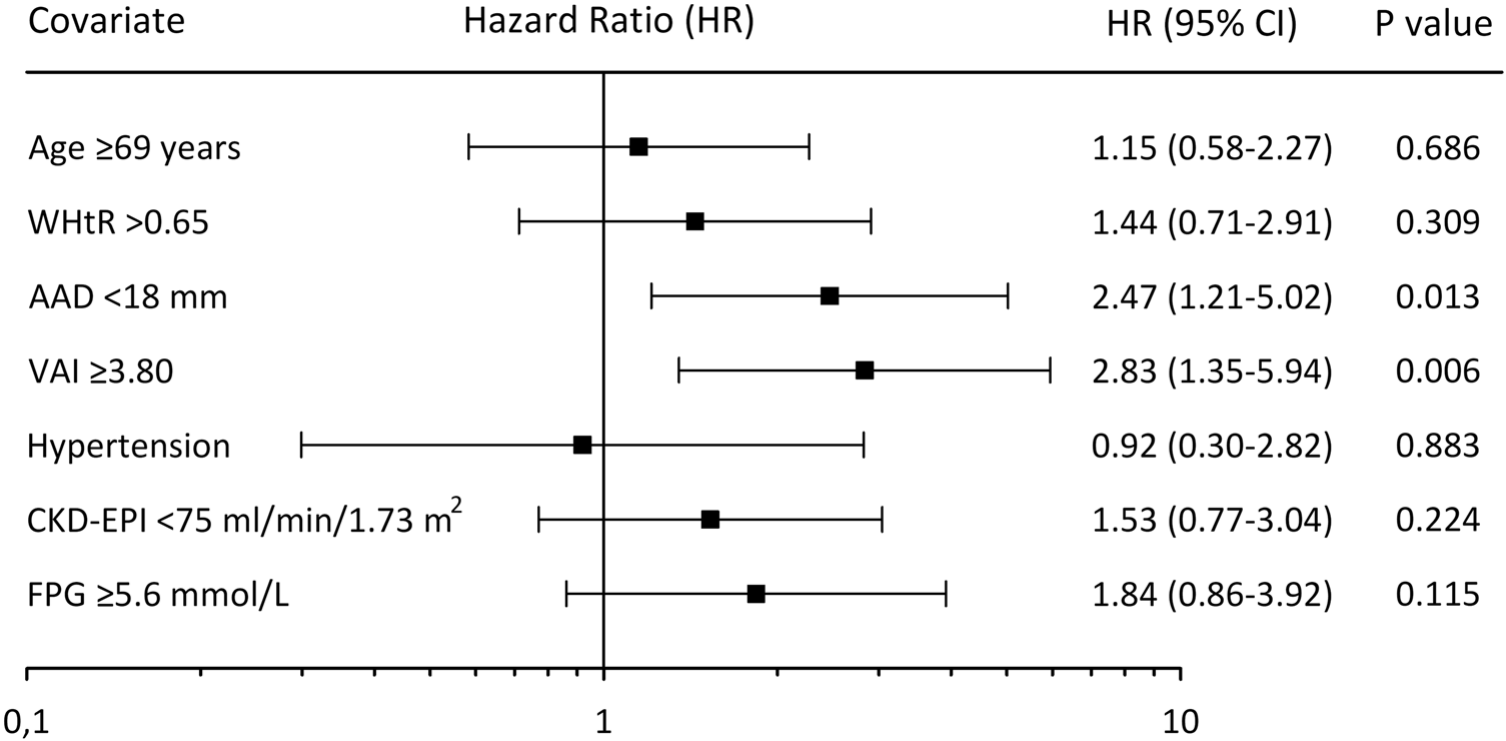
Forest plot of hazard ratios (HR) of diabetes incidence across analyzed variables in the Cox proportional hazard model. *CI* confidence interval, *WHtR* waist-to-height ratio, *AAD* abdominal aorta diameter, *VAI* visceral adiposity index, *FPG* fasting plasma glucose.

Variables significantly related to diabetes incidence risk in the univariate analysis were included in the Cox Proportional Hazard regression model. Due to the strong co-linearity between TG level, TG/HDL ratio and VAI score, we included to this model only VAI, due to its highest HR in the univariate analysis. In addition, we included to this analysis also age, BMI ≥ 25 kg/m^2^ and presence of hypertension, which are established risk factors of diabetes incidence. After validation of different Cox Proportional Hazard models, BMI was removed from the final analysis. In this model only VAI score ≥ 3.80, and AAD < 18 mm appeared to be independently associated with incident diabetes (Fig. 2).

Finally, to determine independent predictors of diabetes incidence, a backward stepwise regression analysis was performed. All potential diabetes risk factors (BMI ≥ 25 kg/m^2^, WC ≥ 88 cm, WHR > 0.85, WHtR > 0.65, AAD < 18 mm, FPG ≥ 5.6 mmol/L, HDL ≤ 1.3 mmol/L, TG ≥ 1.7 mmol/L, TG/HDL ratio ≥ 2.08, VAI score ≥ 3.8, presence of hypertension and eGFR < 75.0 mL/min/1.73 m^2^) were included in this analysis and it was found that diabetes incidence could be predicted from a linear combination of the independent variables: AAD < 18 mm (standardized coefficient β = 0.206, *P* = 0.002), VAI score ≥ 3.80 (β = 0.260, *P* < 0.001) and FPG ≥ 5.6 mmol/L (β = 0.172, *P* = 0.011) (eGFR < 75.0 mL/min/1.73 m^2^ was borderline insignificant, β = 0.129, *P* = 0.053). HR of diabetes development among women with the presence of all these three risk factors was 23-fold higher compared to females without any of them. Women with at least two of these risk factors had a 6.2-fold higher HR of incident diabetes compared to females with no more than one of them (*P* < 0.001 in both analyses).

## Discussion

The actual diabetes incidence in the studied population (2769.2 new cases per 100.000 persons per year) appeared to be apparently higher than expected using National Health Fund and Central Statistical Office data (1860.7 new cases per 100,000 person-years)^4^. However, these differences may arise from the fact that in our study, an active screening was performed in which 11 additional new cases of diabetes were found. Without these cases, diabetes incidence would be 1923.2 per 100,000 person-years, i.e. not far from expectations.

Among analyzed risk factors of diabetes incidence during a 6.5 year follow-up in a group of 200 women aged 65–74 who were non-diabetic at baseline, the only variables independently associated with diabetes risk were VAI and AAD. The same risk factors with addition of FPG ≥ 5.6 mmol/L appeared to be the best predictors of diabetes incidence during this period.

There are many established risk factors of diabetes development^1,5,6^. In the current study conducted in a population of elderly women, WHtR was the only anthropometric marker significantly associated with diabetes incidence in the univariate analysis. However, in the Cox Proportional Hazard regression analysis it lost its significance. Unexpectedly, BMI, WC and WHR were not associated with elevated diabetes incidence risk, probably due to high prevalence of overweight/obesity and abdominal obesity in the studied population. Among single lipid parameters, only elevated TG were significantly related to incident diabetes, while low HDL cholesterol level (≤ 1.3 mmol/L) did not reach the limit of statistical significance. However, two indices including both lipid parameters: VAI and TG/HDL ratio, were significantly associated with elevated risk of diabetes incidence. VAI is considered a reliable predictor of cardio-metabolic risk including diabetes prevalence and incidence^9,15^. In the current study, baseline VAI score ≥ 3.80 was significantly associated with incident diabetes, both in univariate as well as in Cox Proportional Hazard regression model. Moreover, this parameter was a significant predictor of diabetes incidence, together with FPG ≥ 5.6 mmol/L and AAD < 18 mm. Elevated TG/HDL cholesterol ratio is also considered a useful indicator of high cardio-metabolic risk and in several studies appeared to be a predictor of diabetes incidence with different cut-off values depending on ethnicity^16–18^. In the current study, women with baseline TG/HDL ratio ≥ 2.08 had significantly higher diabetes incidence risk in univariate regression analysis. It was not included to the Cox Proportional Hazard model due to the strong co-linearity with VAI score and TG level. Not surprisingly, elevated FPG level at baseline was one of the independent predictors of incident diabetes. Among women with IFG/IGT at baseline, 25 who developed diabetes had significantly lower AAD compared to the remaining 54 females.

Among other established diabetes risk factors, neither hypertension nor blood pressure values were significantly associated with diabetes risk in the current study. The number of patients with a history of cardiovascular events at baseline was too small (n = 15) to perform reliable statistical analysis. Data regarding family history of diabetes mellitus, physical activity, history of gestational diabetes mellitus (GDM), delivering of children with birth weight exceeding 4000 g and polycystic ovary syndrome (PCOS) were not available or were not obtained.

Association of lower abdominal aorta diameter with newly diagnosed diabetes in women at early elderliness was an unexpected finding in our previous study^7^. The current study confirmed this observation and AAD < 18 mm in women aged 65–74 at baseline appears to be significantly and independently associated with diabetes incidence, irrespective of baseline glucose metabolism status. Also in the backward stepwise regression analysis it was, together with VAI score and FPG level, a significant and independent predictor of diabetes incidence. The studies analyzing AAD in this context are very few. Tanaka et al. revealed significantly lower AAD in subjects with DM compared to non-diabetic counterparts in patients with advanced coronary artery disease (CAD)^19^. Inversely, such an association was not found by Taimour et al. in men at age 65 with newly diagnosed diabetes. However, this study was not a prospective observation, and diabetes in the study participants was diagnosed between 2008 and 2015, abdomen ultrasound with AAD measurement was performed between 2010 and 2016, and the authors did not exclude that some patients could be non-diabetic at the time of examination while others could have suffered from diabetes for up to 8 years^20^.

There are some potential mechanisms linking lower AAD with diabetes incidence. It was observed that abdominal aorta aneurysms (AAA) are less prevalent in patients with diabetes compared to non-diabetic subjects^21^. Also, expansion of AAA over time is lower in diabetic patients^22^. One of the postulated mechanisms of this phenomenon is decreased aortic wall extracellular matrix degradation. The extracellular matrix is degraded by matrix metalloproteinases (MMPs). Dua et al. revealed that hyperglycemia is associated with increased expression of plasminogen activator inhibitor-1 (PAI-1), which leads to decreased plasmin generation and, as a result, decreased fibrin degradation and decreased MMPs activity^23^. In an animal model of diabetes, advanced glycation end-products (AGEs) accumulation and AGE-related cross-linking of collagen were responsible for aortic wall matrix stiffness observed in diabetic rats^24^. Nemes et al. observed reduced aortic distensibility during stress transesophageal echocardiography examination in diabetic patients compared to non-diabetic subjects^25^. Åstrand et al. found significantly higher aortic intima-media thickness in diabetic subjects compared to healthy controls^26^. Chirinos et al. revealed increased central pulse pressure, mainly due to impaired arterial compliance in 37 men and women aged 35–55 with type 2 diabetes included in the Asklepios study ($${\raise0.7ex\hbox{$2$} \!\mathord{\left/ {\vphantom {2 3}}\right.\kern-\nulldelimiterspace} \!\lower0.7ex\hbox{$3$}}$$ of them were newly diagnosed)^27^. However, it should be noted that in this study the authors assessed only aortic root and they were unable to detect abnormalities in abdominal aortic diameter^27^. Prenner and Chirinos in their review paper underlined the role of AGEs and nitric oxide dysregulation in the pathogenesis of arterial stiffness not only in overt diabetes, but also in the pre-diabetic stage^28^. These pathomechanisms can explain, at least in part, the relationship between lower AAD and diabetes incidence revealed in our study.

Among limitations of our study, the most important is a relatively small group of study participants. This had an impact on statistical power and it could affect obtained results, especially diabetes incidence rate (although, without patients diagnosed at follow-up visit, it was not far from expectations). Another limitation is lack of data regarding family history of diabetes mellitus, birth weight of the children of study participants, and history of GDM or PCOS. On the other hand, at the time of reproductive age of our study participants (i.e. mainly in the seventies of twentieth century), many of them were delivering at home, assisted by midwife, and precise birth weight of their children were frequently not assessed. Also diagnostic criteria of both GDM and diabetes were different from the current. Moreover, ultrasound examination and many hormone measurements (to diagnose PCOS) were not available at that time in Poland. The last important limitation is fact that our study participants were solely of Caucasian ethnicity, and our results may not be fully applicable to other ethnic groups. However, our study also has strong points. The most important is its prospective design and relatively long follow-up time. Moreover, our study included a representative group of elderly females living in a rural–urban municipality in central Poland, and a wide spectrum of analyzed variables allowed us to identify significant predictors of diabetes incidence in this population, and to find, apart from well-known and established risk factors of diabetes incidence, a novel, potential marker of diabetes risk.

## Conclusions

- Low (< 18 mm) abdominal aorta diameter may be a novel, independent and valuable marker of diabetes risk, if corroborated subsequently in larger studies. In addition, the cut-off points may differ in different ethnic groups. Nevertheless, assessment of abdominal aorta diameter during routine abdomen ultrasound may be helpful in identifying elderly females at high risk of diabetes incidence.
- Visceral adiposity index, containing 4 established diabetes risk factors in its equation, confirmed its usefulness in predicting diabetes and appeared to be better than any of the individual components alone.
- Actual diabetes incidence among women at early elderliness may be higher than expected, and every year roughly 2.8% of them develop diabetes. This indicates the important role of active screening for diabetes in elderly women, especially with risk factors of incident diabetes. However, value of this finding is limited by a small group of study participants.

## Data Availability

The datasets generated and analyzed during the current study are available at the University of Rzeszow Repository under the link: https://repozytorium.ur.edu.pl/handle/item/4895.

## References

[1] International Diabetes Federation. Chapter 3. Global picture. In *IDF Diabetes Atlas* 9th edn. 32–60 (Brussels, International Diabetes Federation, 2019).

[2] Walicka M (2015). Prevalence of diabetes in Poland in the years 2010–2014. Clin. Diabetol..

[3] Sinclair A, Dunning T, Rodriguez-Mañas L (2015). Diabetes in older people: new insights and remaining challenges. Lancet Diabetes Endocrinol..

[4] Ministry of Health. *Map of health needs in the area of diabetes for the Kuyavian-Pomeranian Voivodeship (Mapa potrzeb zdrowotnych w zakresie cukrzycy dla województwa kujawsko-pomorskiego)*. http://www.mpz.mz.gov.pl/wp-content/uploads/sites/4/2018/12/mpz_cukrzyca_kujawsko-pomorskie.pdf (2019).

[5] American Diabetes Association (2019). 2. Classification and diagnosis of diabetes: standards of medical care in diabetes-2019. Diabetes Care.

[6] Diabetes Poland (2019). 2019 Guidelines on the management of diabetic patients. A position of Diabetes Poland. Clin. Diabetol..

[7] Dereziński T, Zozulińska-Ziółkiewicz D, Uruska A, Dąbrowski M (2019). Anthropometric, metabolic and clinical factors associated with diabetes and prediabetes prevalence in women aged 65–74 living in central Poland. Clin. Diabetol..

[8] Central Statistical Office (Główny Urząd Statystyczny) (2013). Demographic Yearbook of Poland 2013 (Rocznik Demograficzny 2013).

[9] Dereziński T, Zozulińska-Ziółkiewicz D, Uruska A, Dąbrowski M (2018). Visceral adiposity index as a useful tool for the assessment of cardio-metabolic risk in women aged 65–74 living in central Poland. Diabetes Metab. Res. Rev..

[10] Touboul PJ (2012). Mannheim carotid intima—media thickness and plaque consensus (2004–2006–2011): an update on behalf of the Advisory Board of the 3rd and 4th Watching the Risk Symposium 13th and 15th European Stroke Conferences, Mannheim, Germany, 2004, and Brussels, Belgium, 2006. Cerebrovasc. Dis..

[11] Levey AS, CKD-EPI (Chronic Kidney Disease Epidemiology Collaboration) (2009). A new equation to estimate glomerular filtration rate. Ann. Intern. Med..

[12] Amato MC, AlkaMeSy Study Group (2010). Visceral adiposity index: a reliable indicator of visceral fat function associated with cardiometabolic risk. Diabetes Care..

[13] World Health Organization (2011). Waist Circumference and Waist-Hip Ratio: Report of a WHO Expert Consultation.

[14] Rogers IS (2013). Distribution, determinants, and normal reference values of thoracic and abdominal aortic diameters by computed tomography (From the Framingham Heart Study). Am. J. Cardiol..

[15] Wang Y (2015). Predictive value of visceral adiposity index for type 2 diabetes mellitus: a 15-year prospective cohort study. Herz.

[16] He S (2012). Higher ratio of triglyceride to high-density lipoprotein cholesterol may predispose to diabetes mellitus: 15-year prospective study in a general population. Metabolism.

[17] Hadaegh F (2010). Lipid ratios and appropriate cut off values for prediction of diabetes: a cohort of Iranian men and women. Lipids Health Dis..

[18] Vega GL, Barlow CE, Grundy SM, Leonard D, DeFina LF (2014). Triglyceride-to-high-density-lipoprotein cholesterol ratio is an index of heart disease mortality and of incidence of type 2 diabetes mellitus in men. J. Investig. Med..

[19] Tanaka A (2015). Inverse association between diabetes and aortic dilatation in patients with advanced coronary artery disease. Atherosclerosis.

[20] Taimour S (2017). Aortic diameter at age 65 in men with newly diagnosed type 2 diabetes. Scand. Cardiovasc. J..

[21] Lederle FA (2012). The strange relationship between diabetes and abdominal aortic aneurysm. Eur. J. Vasc. Endovasc. Surg..

[22] Golledge J (2008). Reduced expansion rate of abdominal aortic aneurysms in patients with diabetes may be related to aberrant monocyte-matrix interactions. Eur. Heart J..

[23] Dua MM (2010). Hyperglycemia modulates plasminogen activator inhibitor-1 expression and aortic diameter in experimental aortic aneurysm disease. Surgery.

[24] Reddy GK (2004). AGE-related cross-linking of collagen is associated with aortic wall matrix stiffness in the pathogenesis of drug-induced diabetes in rats. Microvasc. Res..

[25] Nemes A, Forster T, Lengyel C, Csanády M (2007). Reduced aortic distensibility and coronary flow velocity reserve in diabetes mellitus patients with a negative coronary angiogram. Can. J. Cardiol..

[26] Åstrand H, Rydén-Ahlgren Å, Sundkvist G, Sandgren T, Länne T (2007). Reduced aortic wall stress in diabetes mellitus. Eur. J. Vasc. Endovasc. Surg..

[27] Chirinos JA (2013). Central pulse pressure and its hemodynamic determinants in middle-aged adults with impaired fasting glucose and diabetes: the Asklepios study. Diabetes Care.

[28] Prenner SB, Chirinos JA (2015). Arterial stiffness in diabetes mellitus. Atherosclerosis.

